# Supplementary material for: Common protein-coding variants influence the racing phenotype in galloping racehorse breeds
Source: Commun Biol. 2022 Dec 13;5:1320. doi: 10.1038/s42003-022-04206-x (PMC9748125; doi:10.1038/s42003-022-04206-x)
Supplement: Supplementary file 2 — Supplementary Information [file 42003_2022_4206_MOESM2_ESM.pdf]

SUPPLEMENTARY INFORMATION

Common protein-coding variants influence the racing phenotype in galloping racehorse breeds

Haige Han[1], Beatrice A. McGivney[2], Lucy Allen[3], Dongyi Bai[1], Leanne R. Corduff[2], Gantulga Davaakhuu[4], Jargalsaikhan Davaasambuu[5], Dulguun Dorjgotov[6] , Thomas J. Hall[7], Andrew J. Hemmings[3], Amy R. Holtby[2], Tuyatsetseg Jambal[6], Badarch Jargalsaikhan[8], Uyasakh Jargalsaikhan[5], Naveen K. Kadri[9], David E. MacHugh[7,10], Hubert Pausch[9], Carol Readhead[11], David Warburton[12], Manglai Dugarjaviin[1,*] and Emmeline W. Hill[2,7,*]

Supplementary Note 1: Biological functions of genes chosen for validation genotyping

Muscle: The protein encoded by *ANKRD23* (ankyrin repeat domain 23) functions as a molecular link between the stretch-induced mechanical stimulus and skeletal muscle gene expression in response to exercise [1-3]; *HDAC9* (histone deacetylase 9) encodes a protein that inhibits skeletal myogenesis and is involved in heart development [4-7]; *MYLK2* (myosin light chain kinase 2) is expressed in skeletal muscle and its product functions in muscle contraction, neuromuscular synaptic transmission, skeletal muscle satellite cell differentiation, regulation of muscle filament sliding, skeletal muscle cell differentiation, cardiac muscle tissue morphogenesis, and cardiac muscle contraction [8-12]; myomesin 2, encoded by *MYOM2* (myomesin 2), is a major component of the vertebrate myofibrillar M band that binds myosin, titin, and light meromyosin and is involved in muscle contraction [13-15];

Heart: The protein encoded by *ATP1A1* (ATPase Na+/K+ transporting subunit alpha 1) regulates the force of heart contraction [16,17]; the CXADR Ig-like cell adhesion molecule protein, encoded by *CXADR* (CXADR Ig-like cell adhesion molecule), functions in heart development and is associated with arrhythmia [18-20]; the *PPP2R3A* (protein phosphatase 2 regulatory subunit B"alpha) gene product is required for cardiac development and is associated with cardiac disease [21,22];

Angiogenesis / blood: *HMOX1* (heme oxygenase 1) encodes an enzyme essential for heme catabolism, with activity highest in the spleen [23-28]; the product of *VEGFA* (vascular

endothelial growth factor A) is a growth factor that induces proliferation and migration of vascular endothelial cells, essential for both physiological and pathological angiogenesis [29-31];

Limb development: Cartilage oligomeric matrix protein, encoded by *COMP* (cartilage oligomeric matrix protein), plays a major role in the structural integrity of cartilage, is used to predict tendon damage in horses, and is a biomarker for equine osteoarthritis [32-36]; the *INTU* (inturned planar cell polarity protein) gene product is involved in limb development and *Intu* mouse mutants exhibit rib defects and endochondral ossification delay [37]; the T-box transcription factor 15 protein, encoded by *TBX15* (T-Box transcription factor 15), is involved in skeletal development of the limb, vertebral column and shoulder and pelvic girdles and also functions in skeletal muscle metabolism [38-43];

Metabolism: The product of the *CRB4* (carbonyl reductase 4) gene functions in mitochondrial fatty acid biosynthesis [44-46]; the FAST kinase domains 1 protein, encoded by *FASTKD1* (FAST kinase domains 1), *s*upports mitochondrial homeostasis and has a critical protective role against oxidant-induced cell death [47-50]; *G6PC2* (glucose-6-phosphatase catalytic subunit 2) encodes a major component of glycolysis [51-53]; the protein encoded by *GLB1* (galactosidase beta 1) has a role in number of metabolic pathways and is the most widely used biomarker for senescent and aging cells [54]; lipin 1, encoded by *LPIN1* (lipin 1), is involved in glycerolipid metabolism [55-61]; *PKM* (pyruvate kinase M1/2) encodes an important protein involved in glycolysis [62,63]; the solute carrier family 16 member 1 protein, encoded by *SLC16A1* (solute carrier family 16 member 1), catalyses the movement of lactate and pyruvate across the plasma membrane [64-66];

Neurological: The product of *KPNA3* (karyopherin subunit alpha 3) is associated with neurobiological defects [67-69]; an eQTL associated with *KTN1* (kinectin 1) gene expression in the frontal cortex is strongly associated with putamen volume in the brain, which influences motor behaviours including motor planning and execution, motor preparation, amplitudes of movement and sequences of movement [70-77]; neurotrimin, encoded by *NTM* (neurotrimin), functions in brain development, regulates neural growth and synapse formation and influences learning and memory [78-83]; *NPY* (neuropeptide Y) is widely expressed in the central nervous system influencing many physiological processes, including cortical excitability, stress response, food intake, circadian rhythms, cardiovascular function and response to chronic pain [84-90]; prolylcarboxypeptidase. encoded by *PRCP* (prolylcarboxypeptidase), is involved in the interaction between voluntary exercise and body composition and in Thoroughbreds variants at the locus are associated with race starts [91-93]; *SULT4A1* (sulfotransferase family 4A member 1) encodes a brain-specific sulfotransferase involved in the metabolism of neurotransmitters [94-

[96]; the product of the *SYNDIG1* (synapse differentiation inducing 1) gene regulates the development of excitatory synapses [97-99].

Supplementary Note 2: Horse breeds

Thoroughbreds are the most intensively selected horse population for racing performance and are extensively managed in highly controlled husbandry environments with breeding decisions undertaken as high-value investments. Thoroughbreds are geographically widespread, though there is considerable gene flow between regions, especially the major breeding and racing hubs in North America, Europe, Australasia, and Japan. Fewer than 8% of horses achieve elite racing status, which is generally defined as winning at the highest national or international level in Graded or Group races.

Present-day Mongolian horses are found in northeast and north China (mainly Inner Mongolia), Mongolia, and some areas of eastern Russia. Approximately three million horses occupy a vast geographical space across Mongolia and Inner Mongolia, China. These animals experience extreme environmental and climatic conditions with minimal human intervention or systematic breeding [100,101]. Racing herds, however, are highly prized and while they are mostly free ranging, there is some ongoing selection by herdsmen for racing attributes based on performance in regional and national races.

Arabian horses originated in the Middle East and now comprise several geographical and phenotypic subgroups [102]. Arabians are used both in racing and sport. In sport, Arabian horses excel in endurance racing competitions, one of the six sports managed by the international governing body for equestrian sports, Fédération Équestre Internationale (FEI; fei.org). In this study, Arabian horses were included among the Racing cohort for the purposes of identifying selection signals for racing. They were not used for further downstream analyses. Increasing the number of samples from breeds with similar phenotypes can increase the opportunity to detect selection signals when the contrasting phenotypes are very different [103]. Since we had no additional phenotype information for the Arabian horses used in this study, we performed a principle component analysis using our samples (n = 30 ARR) along with Arabian horses with confirmed competition use (n = 23 endurance, n = 29 racing and n = 100 show) according to Cosgrove *et al.* [102]. The Arabian horses (ARR) used in this study were distributed across the variation for the Arabian horses used for racing (Supplementary Figure 7). Therefore, the Arabian population used in this study to detect selection signals for racing is confirmed to represent the racing phenotype. The Arabian samples used in this study were

submitted for genetic testing from the United Arab Emirates. No additional pedigree information was available. Therefore, we cannot be certain that the horses fulfil the requirement of the World Arabian Horse Organisation (WAHO) for the definition of a Purebred Arabian that states: "A Purebred Arabian horse is one which appears in any purebred Arabian Stud Book or Register listed by WAHO as acceptable". Since the samples fall within the genetic variation for other Arabian horses in the public domain, throughout the manuscript the population is referred to simply as Arabian, as it is uncertain that they fulfil the definition of 'Purebred Arabian'.

For the SNP association tests, among the racing breeds, two were trotting breeds (Standardbred and French Trotter) and although selected for a different type of racing there was some overlap between the Racing selection signals identified in this study and targets of selection previously identified in Standardbred. A subpopulation of the Quarter Horse is used for galloping racing and the breed gets its name from excelling in short distance sprinting. Here, no phenotypic or subpopulation assignment was available for the Quarter Horse samples, so while they were included among the racing breeds, it is not certain that any or all of them were active racehorses. Among the non-racing breeds, five were Chinese Mongolian landrace breeds (Baerhu, Baicha Iron Hoof, Keerqin, Wushen, and Wuzhumuqin), three were putative ancestral populations contributing to the Thoroughbred (Akhal Teke, Egyptian Arabian, and Moroccan Barb), and three were sport horse breeds (Connemara, Irish Draught, and Dutch Warmblood).

Supplementary Figures

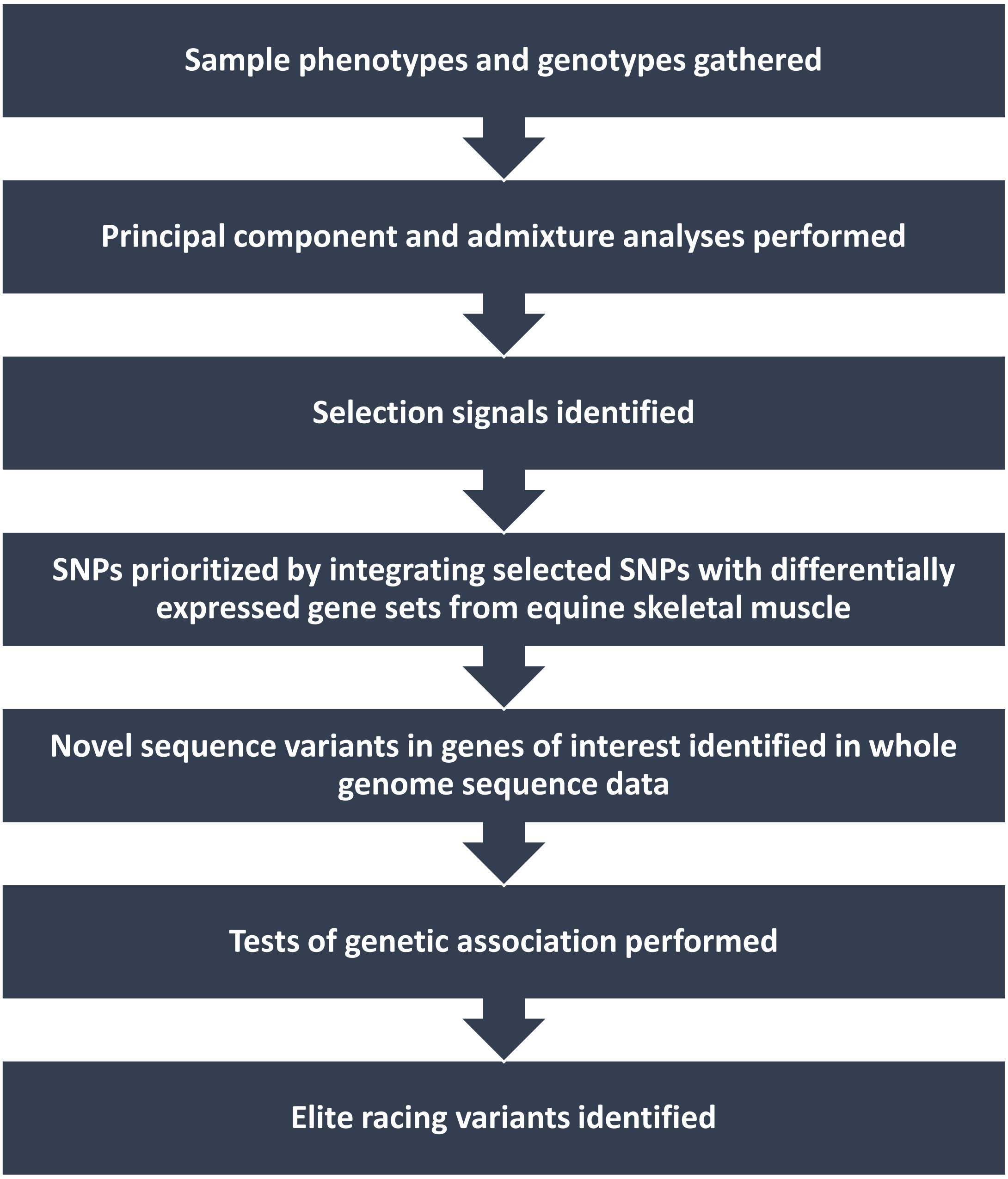

Supplementary Figure 1: Flow chart showing sequential steps of the study

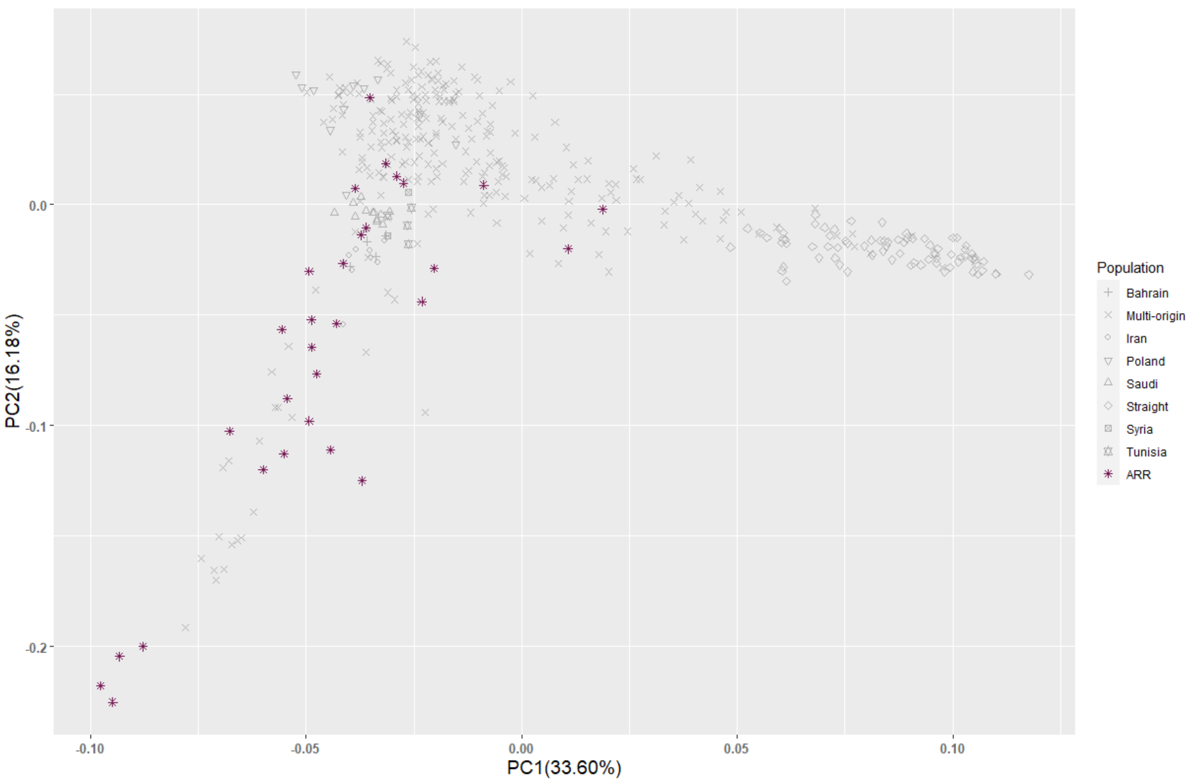

Supplementary Figure 2. PCA plot for 408 Arabian horses using 35,292 genome-wide SNPs. Arabian (ARR) horses genotyped in this study are highlighted (purple).

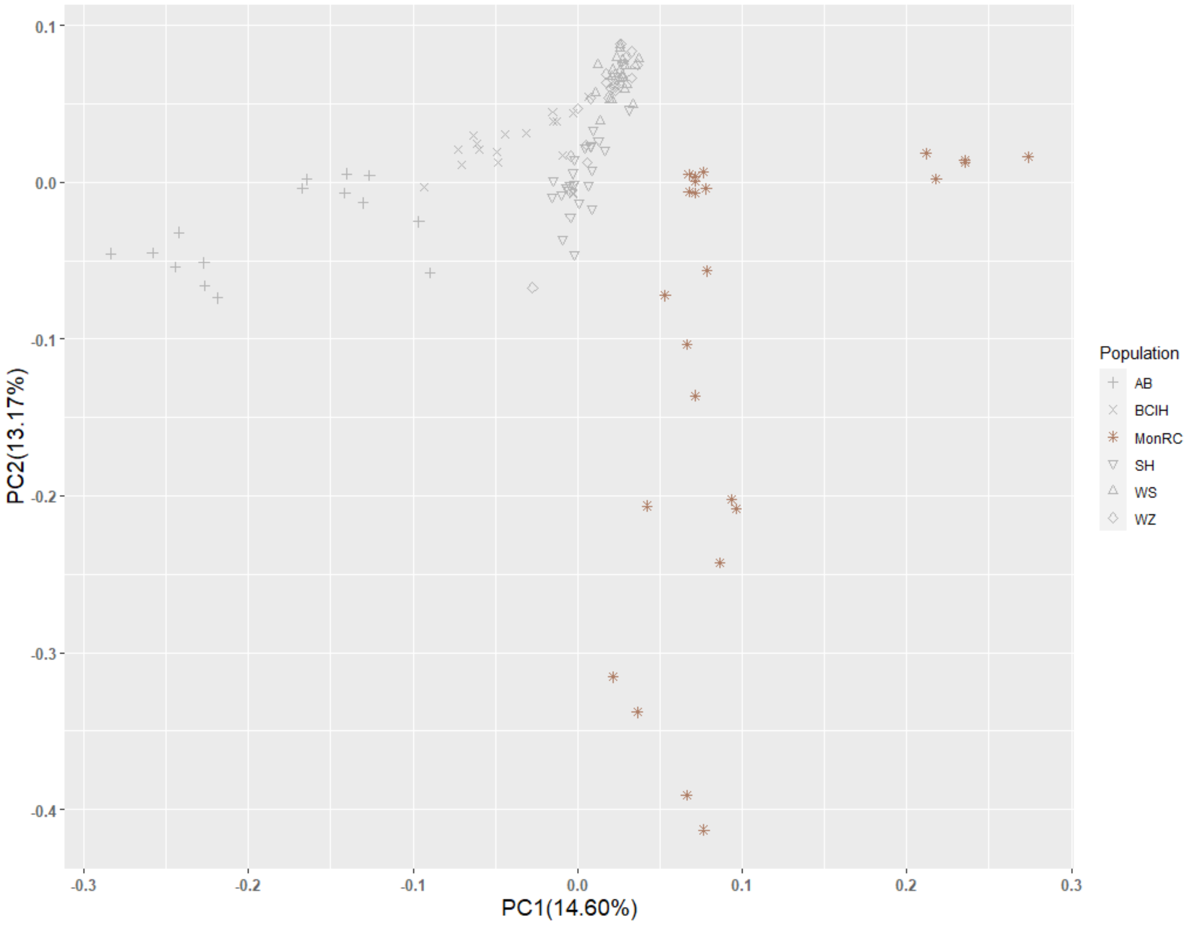

Supplementary Figure 3: PCA plot for 124 Mongolian horses using 60,994 genome-wide SNPs. Racing Mongolian (MonR) horses genotyped in this study are highlighted (brown).

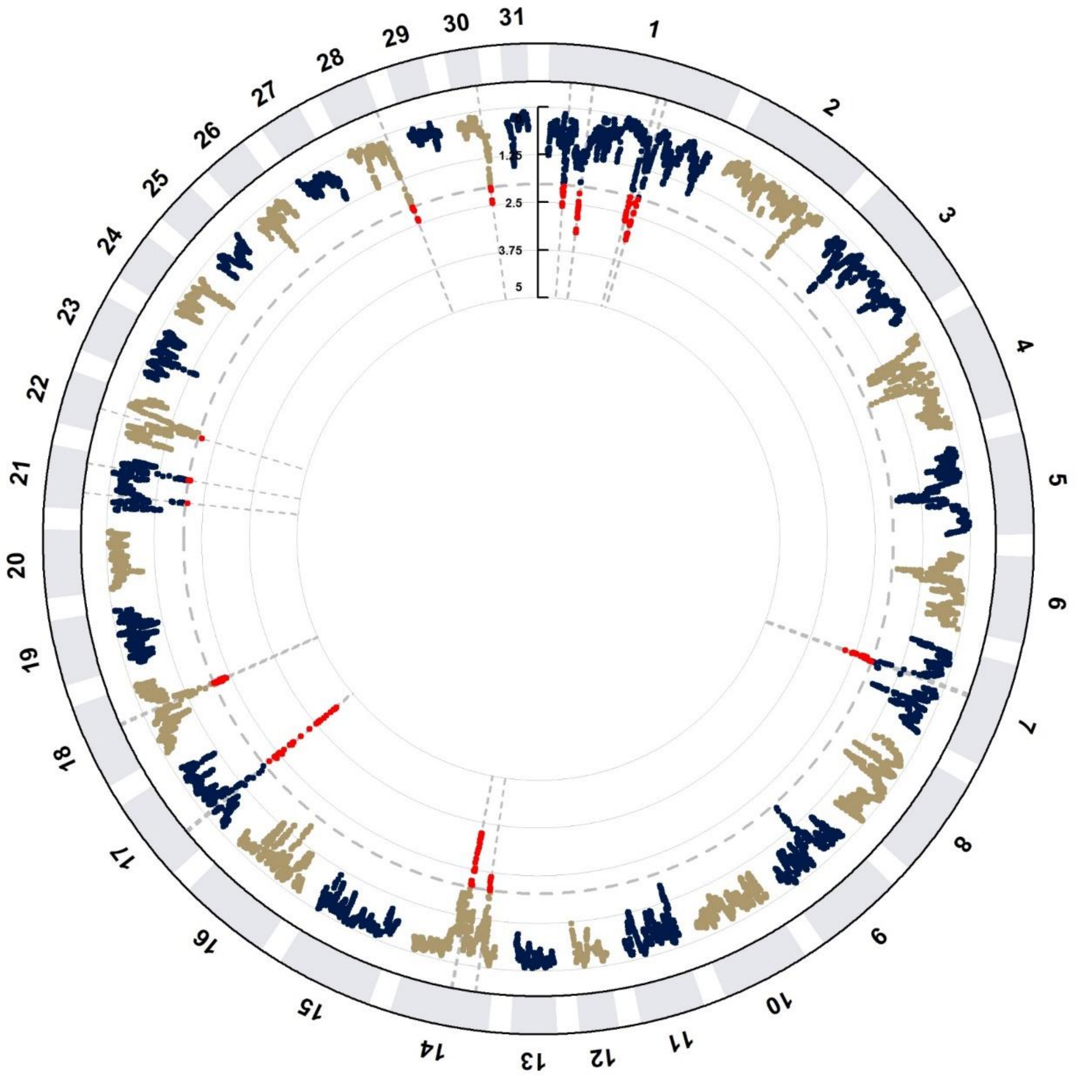

Supplementary Figure 4: Manhattan plot for the results of the composite selection signals (CSS) analysis to detect targets of selection among Thoroughbred (TB) horses when compared with other breeds. The results were obtained by averaging the CSS scores of SNPs within 100 kb sliding windows. The dashed grey line indicates the genome-wide (1% SNPs) threshold of the empirical scores and the top SNPs are indicated by red dots.

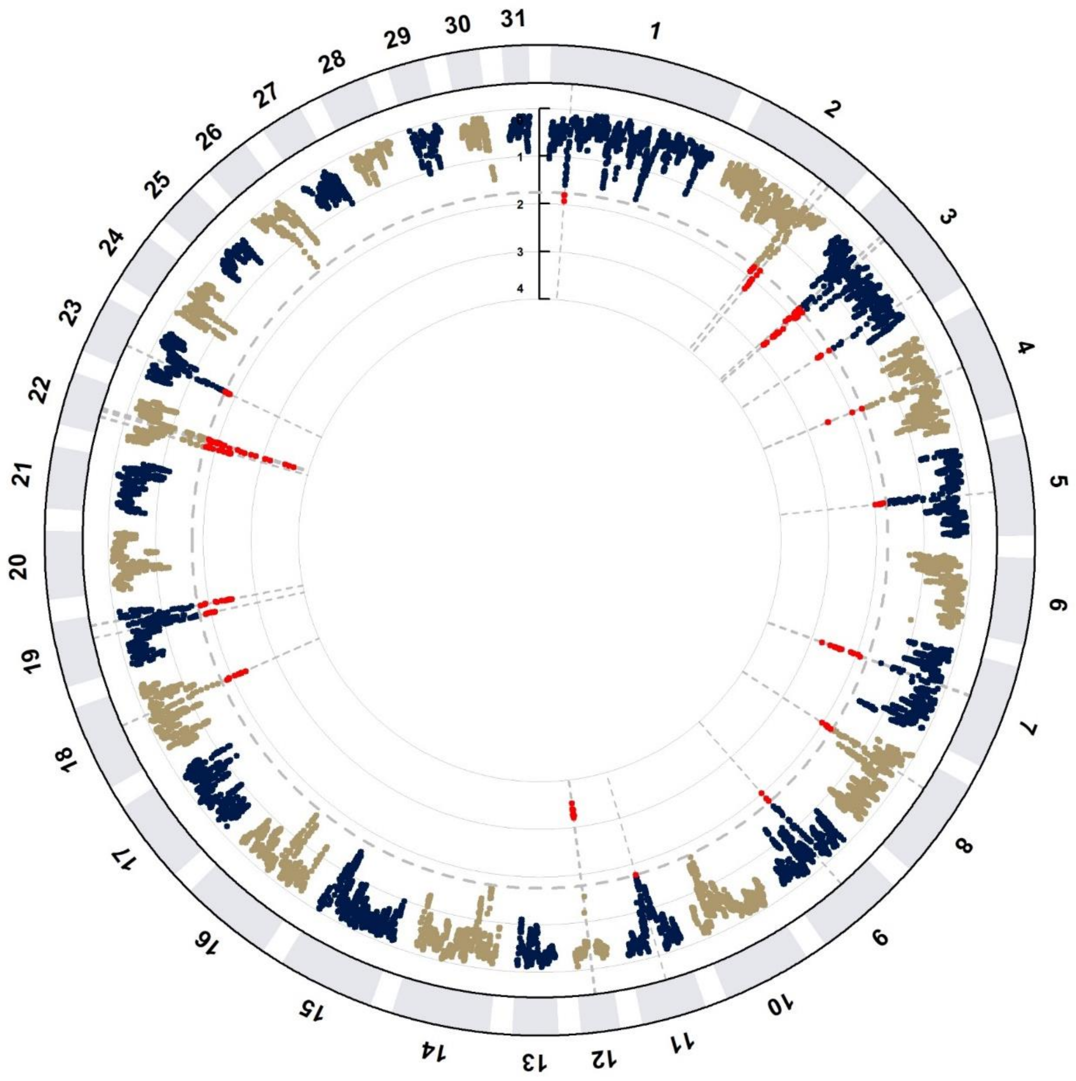

Supplementary Figure 5: Manhattan plot for the results of the composite selection signals (CSS) analysis to detect targets of selection among Arabian (ARR) horses when compared with other breeds. The results were obtained by averaging the CSS scores of SNPs within 100 kb sliding windows. The dashed grey line indicates the genome-wide (1% SNPs) threshold of the empirical scores and the top SNPs are indicated by red dots.

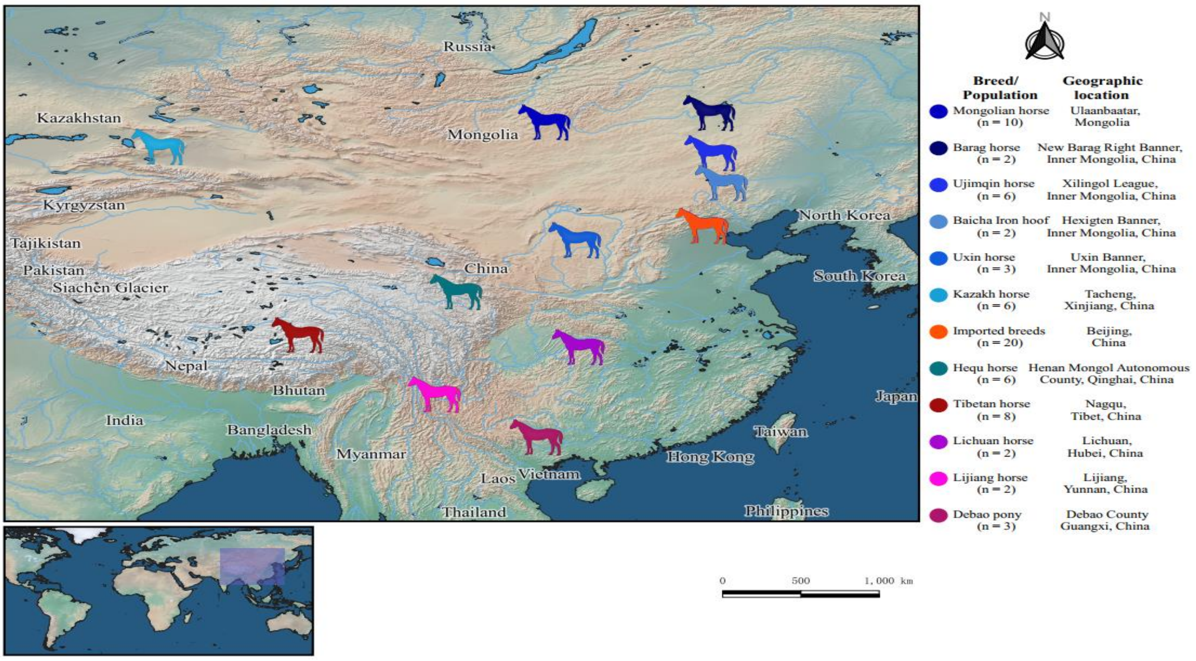

Supplementary Figure 6: Samples and population details for 70 horses used for whole genome resequencing. Map graphics were created using the free and open-source QGIS software package [104].

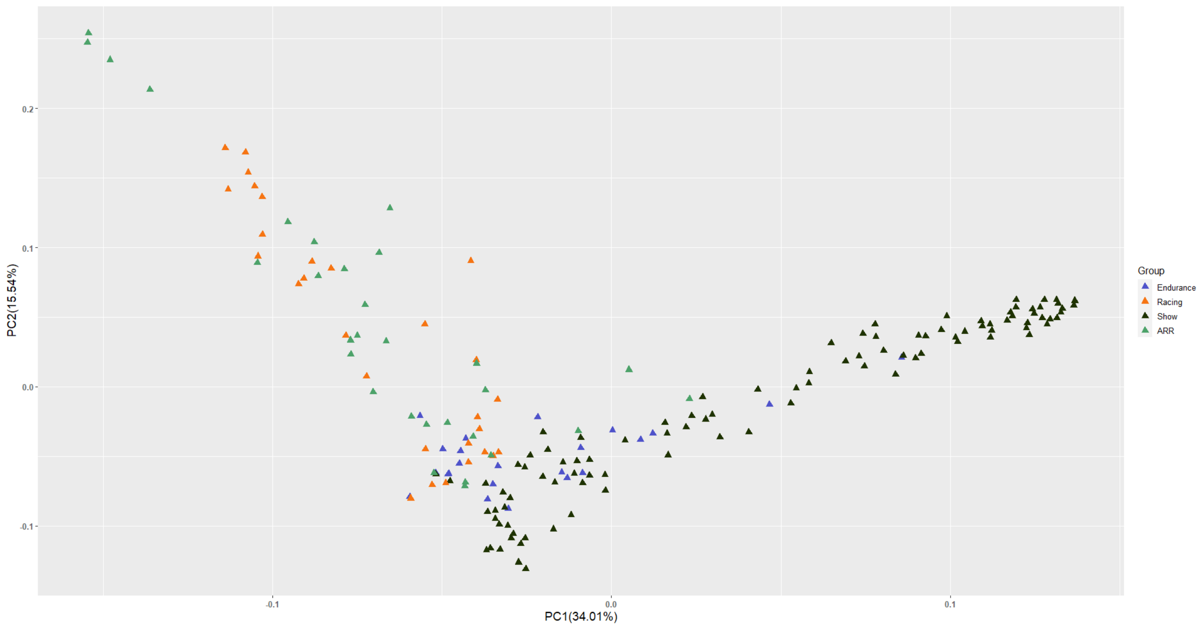

Supplementary Figure 7: PCA plot for 182 Arabian horses colour coded by competition use. Arabian (ARR) horses genotyped in this study (green) are predominantly distributed in the PCA space that includes the Racing horses (orange).

Supplementary References

1 Shimoda, Y. *et al.* Diabetes-Related Ankyrin Repeat Protein (DARP/Ankrd23) Modifies Glucose Homeostasis by Modulating AMPK Activity in Skeletal Muscle. *PLoS One* **10**, e0138624, doi:10.1371/journal.pone.0138624 (2015).

2 Laughlin, M. H. *et al.* Vascular cell transcriptomic changes to exercise training differ directionally along and between skeletal muscle arteriolar trees. *Microcirculation* **24**, doi:10.1111/micc.12336 (2017).

3 Wang, X., Zeng, R., Xu, H., Xu, Z. & Zuo, B. The nuclear protein-coding gene ANKRD23 negatively regulates myoblast differentiation. *Gene* **629**, 68-75, doi:10.1016/j.gene.2017.07.062 (2017).

4 Macpherson, P. C., Farshi, P. & Goldman, D. Dach2-Hdac9 signaling regulates reinnervation of muscle endplates. *Development* **142**, 4038-4048, doi:10.1242/dev.125674 (2015).

5 Mejat, A. *et al.* Histone deacetylase 9 couples neuronal activity to muscle chromatin acetylation and gene expression. *Nat Neurosci* **8**, 313-321, doi:10.1038/nn1408 (2005).

6 Malhotra, R. *et al.* HDAC9 is implicated in atherosclerotic aortic calcification and affects vascular smooth muscle cell phenotype. *Nat Genet* **51**, 1580-1587, doi:10.1038/s41588-019-0514-8 (2019).

7 Hu, S., Cho, E. H. & Lee, J. Y. Histone Deacetylase 9: Its Role in the Pathogenesis of Diabetes and Other Chronic Diseases. *Diabetes Metab J* **44**, 234-244, doi:10.4093/dmj.2019.0243 (2020).

8 Zhi, G. *et al.* Myosin light chain kinase and myosin phosphorylation effect frequency-dependent potentiation of skeletal muscle contraction. *Proc Natl Acad Sci U S A* **102**, 17519-17524, doi:10.1073/pnas.0506846102 (2005).

9 Stull, J. T., Kamm, K. E. & Vandenboom, R. Myosin light chain kinase and the role of myosin light chain phosphorylation in skeletal muscle. *Arch Biochem Biophys* **510**, 120-128, doi:10.1016/j.abb.2011.01.017 (2011).

10 Sheikh, F., Lyon, R. C. & Chen, J. Functions of myosin light chain-2 (MYL2) in cardiac muscle and disease. *Gene* **569**, 14-20, doi:10.1016/j.gene.2015.06.027 (2015).

11 Alvarez-Santos, M. D., Alvarez-Gonzalez, M., Estrada-Soto, S. & Bazan-Perkins, B. Regulation of Myosin Light-Chain Phosphatase Activity to Generate Airway Smooth Muscle Hypercontractility. *Front Physiol* **11**, 701, doi:10.3389/fphys.2020.00701 (2020).

12 Qin, X. *et al.* FLNC and MYLK2 Gene Mutations in a Chinese Family with Different Phenotypes of Cardiomyopathy. *Int Heart J* **62**, 127-134, doi:10.1536/ihj.20-351 (2021).

13 Sweeney, H. L. & Hammers, D. W. Muscle Contraction. *Cold Spring Harb Perspect Biol* **10**, doi:10.1101/cshperspect.a023200 (2018).

14 Pehlivan, D. *et al.* The Genomics of Arthrogryposis, a Complex Trait: Candidate Genes and Further Evidence for Oligogenic Inheritance. *Am J Hum Genet* **105**, 132-150, doi:10.1016/j.ajhg.2019.05.015 (2019).

15 Auxerre-Plantie, E. *et al.* Identification of MYOM2 as a candidate gene in hypertrophic cardiomyopathy and Tetralogy of Fallot, and its functional evaluation in the Drosophila heart. *Dis Model Mech* **13**, doi:10.1242/dmm.045377 (2020).

16 He, J. *et al.* ATP1A1 mutations cause intermediate Charcot-Marie-Tooth disease. *Hum Mutat* **40**, 2334-2343, doi:10.1002/humu.23886 (2019).

17 Aughey, R. J. *et al.* Muscle Na+-K+-ATPase activity and isoform adaptations to intense interval exercise and training in well-trained athletes. *J Appl Physiol (1985)* **103**, 39-47, doi:10.1152/japplphysiol.00236.2006 (2007).

18 Marsman, R. F. *et al.* Coxsackie and adenovirus receptor is a modifier of cardiac conduction and arrhythmia vulnerability in the setting of myocardial ischemia. *J Am Coll Cardiol* **63**, 549-559, doi:10.1016/j.jacc.2013.10.062 (2014).

19 Chopra, N. & Knollmann, B. C. Genetics of sudden cardiac death syndromes. *Curr Opin Cardiol* **26**, 196-203, doi:10.1097/HCO.0b013e3283459893 (2011).

20 Bezzina, C. R. *et al.* Genome-wide association study identifies a susceptibility locus at 21q21 for ventricular fibrillation in acute myocardial infarction. *Nat Genet* **42**, 688-691, doi:10.1038/ng.623 (2010).
21 Song, G. *et al.* Deletion of Pr72 causes cardiac developmental defects in Zebrafish. *PLoS One* **13**, e0206883, doi:10.1371/journal.pone.0206883 (2018).
22 Li, M. *et al.* Discovery of PPP2R3A and TMX3 pathogenic variants in a Zhuang family with coronary artery disease using whole-exome sequencing. *Int J Clin Exp Pathol* **11**, 3678-3684 (2018).
23 Seidel, U., Huebbe, P. & Rimbach, G. Taurine: A Regulator of Cellular Redox Homeostasis and Skeletal Muscle Function. *Mol Nutr Food Res* **63**, e1800569, doi:10.1002/mnfr.201800569 (2019).
24 Pietraszek-Gremplewicz, K. *et al.* Heme Oxygenase-1 Influences Satellite Cells and Progression of Duchenne Muscular Dystrophy in Mice. *Antioxid Redox Signal* **29**, 128-148, doi:10.1089/ars.2017.7435 (2018).
25 Park, C. H. *et al.* Hemin, heme oxygenase-1 inducer, attenuates immobilization-induced skeletal muscle atrophy in mice. *Life Sci* **92**, 740-746, doi:10.1016/j.lfs.2013.02.008 (2013).
26 Vesely, M. J. *et al.* Heme oxygenase-1 induction in skeletal muscle cells: hemin and sodium nitroprusside are regulators in vitro. *Am J Physiol* **275**, C1087-1094, doi:10.1152/ajpcell.1998.275.4.C1087 (1998).
27 Varga, C. *et al.* The Effects of Exercise Training and High Triglyceride Diet in an Estrogen Depleted Rat Model: The Role of the Heme Oxygenase System and Inflammatory Processes in Cardiovascular Risk. *J Sports Sci Med* **17**, 580-588 (2018).
28 Fraser, S. T. *et al.* Heme oxygenase-1 deficiency alters erythroblastic island formation, steady-state erythropoiesis and red blood cell lifespan in mice. *Haematologica* **100**, 601-610, doi:10.3324/haematol.2014.116368 (2015).
29 Bloor, C. M. Angiogenesis during exercise and training. *Angiogenesis* **8**, 263-271, doi:10.1007/s10456-005-9013-x (2005).
30 Gustafsson, T. Vascular remodelling in human skeletal muscle. *Biochem Soc Trans* **39**, 1628-1632, doi:10.1042/BST20110720 (2011).
31 Chen, L., Bai, J. & Li, Y. miR29 mediates exerciseinduced skeletal muscle angiogenesis by targeting VEGFA, COL4A1 and COL4A2 via the PI3K/Akt signaling pathway. *Mol Med Rep* **22**, 661-670, doi:10.3892/mmr.2020.11164 (2020).
32 Smith, M. R. *et al.* Increased cartilage oligomeric matrix protein concentrations in equine digital flexor tendon sheath synovial fluid predicts intrathecal tendon damage. *Vet Surg* **40**, 54-58, doi:10.1111/j.1532-950X.2010.00751.x (2011).
33 Skioldebrand, E. *et al.* Cartilage oligomeric matrix protein neoepitope in the synovial fluid of horses with acute lameness: A new biomarker for the early stages of osteoarthritis. *Equine Vet J* **49**, 662-667, doi:10.1111/evj.12666 (2017).
34 Smith, R. K. & Heinegard, D. Cartilage oligomeric matrix protein (COMP) levels in digital sheath synovial fluid and serum with tendon injury. *Equine Vet J* **32**, 52-58, doi:10.2746/042516400777612053 (2000).
35 Misumi, K. *et al.* Urine cartilage oligomeric matrix protein (COMP) measurement is useful in discriminating the osteoarthritic Thoroughbreds. *Osteoarthritis Cartilage* **14**, 1174-1180, doi:10.1016/j.joca.2006.04.017 (2006).
36 Skioldebrand, E., Ekman, S., Heinegard, D. & Hultenby, K. Ultrastructural immunolocalization of cartilage oligomeric matrix protein (COMP) in the articular cartilage on the equine third carpal bone in trained and untrained horses. *Res Vet Sci* **88**, 251-257, doi:10.1016/j.rvsc.2009.07.011 (2010).
37 Chang, R., Petersen, J. R., Niswander, L. A. & Liu, A. A hypomorphic allele reveals an important role of inturned in mouse skeletal development. *Dev Dyn* **244**, 736-747, doi:10.1002/dvdy.24272 (2015).

38 Singh, M. K. *et al.* The T-box transcription factor Tbx15 is required for skeletal development. *Mech Dev* **122**, 131-144, doi:10.1016/j.mod.2004.10.011 (2005).
39 Kuijper, S. *et al.* Genetics of shoulder girdle formation: roles of Tbx15 and aristaless-like genes. *Development* **132**, 1601-1610, doi:10.1242/dev.01735 (2005).
40 Lee, K. Y. *et al.* Tbx15 Defines a Glycolytic Subpopulation and White Adipocyte Heterogeneity. *Diabetes* **66**, 2822-2829, doi:10.2337/db17-0218 (2017).
41 Lee, K. Y. *et al.* Tbx15 controls skeletal muscle fibre-type determination and muscle metabolism. *Nat Commun* **6**, 8054, doi:10.1038/ncomms9054 (2015).
42 Gesta, S. *et al.* Evidence for a role of developmental genes in the origin of obesity and body fat distribution. *Proc Natl Acad Sci U S A* **103**, 6676-6681, doi:10.1073/pnas.0601752103 (2006).
43 Sun, W. *et al.* Tbx15 is required for adipocyte browning induced by adrenergic signaling pathway. *Mol Metab* **28**, 48-57, doi:10.1016/j.molmet.2019.07.004 (2019).
44 Cai, C. *et al.* Comparative Transcriptome Analyses of Longissimus thoracis Between Pig Breeds Differing in Muscle Characteristics. *Front Genet* **11**, 526309, doi:10.3389/fgene.2020.526309 (2020).
45 Chen, Z. *et al.* 17beta-hydroxysteroid dehydrogenase type 8 and carbonyl reductase type 4 assemble as a ketoacyl reductase of human mitochondrial FAS. *FASEB J* **23**, 3682-3691, doi:10.1096/fj.09-133587 (2009).
46 Venkatesan, R. *et al.* Insights into mitochondrial fatty acid synthesis from the structure of heterotetrameric 3-ketoacyl-ACP reductase/3R-hydroxyacyl-CoA dehydrogenase. *Nat Commun* **5**, 4805, doi:10.1038/ncomms5805 (2014).
47 Boehm, E. *et al.* Role of FAST Kinase Domains 3 (FASTKD3) in Post-transcriptional Regulation of Mitochondrial Gene Expression. *J Biol Chem* **291**, 25877-25887, doi:10.1074/jbc.M116.730291 (2016).
48 Marshall, K. D., Klutho, P. J., Song, L., Krenz, M. & Baines, C. P. The novel cyclophilin-D-interacting protein FASTKD1 protects cells against oxidative stress-induced cell death. *Am J Physiol Cell Physiol* **317**, C584-C599, doi:10.1152/ajpcell.00471.2018 (2019).
49 Jourdain, A. A. *et al.* The FASTK family of proteins: emerging regulators of mitochondrial RNA biology. *Nucleic Acids Res* **45**, 10941-10947, doi:10.1093/nar/gkx772 (2017).
50 Simarro, M. *et al.* Fast kinase domain-containing protein 3 is a mitochondrial protein essential for cellular respiration. *Biochem Biophys Res Commun* **401**, 440-446, doi:10.1016/j.bbrc.2010.09.075 (2010).
51 Dupuis, J. *et al.* New genetic loci implicated in fasting glucose homeostasis and their impact on type 2 diabetes risk. *Nat Genet* **42**, 105-116, doi:10.1038/ng.520 (2010).
52 Bouatia-Naji, N. *et al.* A polymorphism within the G6PC2 gene is associated with fasting plasma glucose levels. *Science* **320**, 1085-1088, doi:10.1126/science.1156849 (2008).
53 Mahajan, A. *et al.* Identification and functional characterization of G6PC2 coding variants influencing glycemic traits define an effector transcript at the G6PC2-ABCB11 locus. *PLoS Genet* **11**, e1004876, doi:10.1371/journal.pgen.1004876 (2015).
54 Dimri, G. P. *et al.* A biomarker that identifies senescent human cells in culture and in aging skin in vivo. *Proc Natl Acad Sci U S A* **92**, 9363-9367, doi:10.1073/pnas.92.20.9363 (1995).
55 Han, B. *et al.* Genetic Effects of LPIN1 Polymorphisms on Milk Production Traits in Dairy Cattle. *Genes (Basel)* **10**, doi:10.3390/genes10040265 (2019).
56 Che, R. *et al.* A rare case of pediatric recurrent rhabdomyolysis with compound heterogenous variants in the LPIN1. *BMC Pediatr* **20**, 218, doi:10.1186/s12887-020-02134-5 (2020).
57 Pizzamiglio, C. *et al.* First presentation of LPIN1 acute rhabdomyolysis in adolescence and adulthood. *Neuromuscul Disord* **30**, 566-571, doi:10.1016/j.nmd.2020.05.004 (2020).
58 Legendre, A. *et al.* Cardiac function and exercise adaptation in 8 children with LPIN1 mutations. *Mol Genet Metab* **123**, 375-381, doi:10.1016/j.ymgme.2017.12.429 (2018).

59 Fu, S. *et al.* Transcriptomic Responses of Skeletal Muscle to Acute Exercise in Diabetic Goto-Kakizaki Rats. *Front Physiol* **10**, 872, doi:10.3389/fphys.2019.00872 (2019).
60 Michot, C. *et al.* Study of LPIN1, LPIN2 and LPIN3 in rhabdomyolysis and exercise-induced myalgia. *J Inherit Metab Dis* **35**, 1119-1128, doi:10.1007/s10545-012-9461-6 (2012).
61 Raaschou-Pedersen, D. *et al.* Fat oxidation is impaired during exercise in lipin-1 deficiency. *Neurology* **93**, e1433-e1438, doi:10.1212/WNL.0000000000008240 (2019).
62 Zhang, Z. *et al.* PKM2, function and expression and regulation. *Cell Biosci* **9**, 52, doi:10.1186/s13578-019-0317-8 (2019).
63 Verbrugge, S. A. J. *et al.* PKM2 Determines Myofiber Hypertrophy In Vitro and Increases in Response to Resistance Exercise in Human Skeletal Muscle. *Int J Mol Sci* **21**, doi:10.3390/ijms21197062 (2020).
64 Halestrap, A. P. The SLC16 gene family - structure, role and regulation in health and disease. *Mol Aspects Med* **34**, 337-349, doi:10.1016/j.mam.2012.05.003 (2013).
65 Ropka-Molik, K., Stefaniuk-Szmukier, M., Szmatola, T., Piorkowska, K. & Bugno-Poniewierska, M. The use of the SLC16A1 gene as a potential marker to predict race performance in Arabian horses. *BMC Genet* **20**, 73, doi:10.1186/s12863-019-0774-4 (2019).
66 Fontanel, M. *et al.* Variation in the SLC16A1 and the ACOX1 Genes Is Associated with Gallop Racing Performance in Arabian Horses. *J Equine Vet Sci* **93**, 103202, doi:10.1016/j.jevs.2020.103202 (2020).
67 Morris, C. P. *et al.* KPNA3 variation is associated with schizophrenia, major depression, opiate dependence and alcohol dependence. *Dis Markers* **33**, 163-170, doi:10.3233/DMA-2012-0921 (2012).
68 Sowa, A. S. *et al.* Karyopherin alpha-3 is a key protein in the pathogenesis of spinocerebellar ataxia type 3 controlling the nuclear localization of ataxin-3. *Proc Natl Acad Sci U S A* **115**, E2624-E2633, doi:10.1073/pnas.1716071115 (2018).
69 Xie, L. *et al.* Genome-wide association study identified a narrow chromosome 1 region associated with chicken growth traits. *PLoS One* **7**, e30910, doi:10.1371/journal.pone.0030910 (2012).
70 Zhang, Y. *et al.* Differential expression profiling between the relative normal and dystrophic muscle tissues from the same LGMD patient. *J Transl Med* **4**, 53, doi:10.1186/1479-5876-4-53 (2006).
71 Abe, E., Okawa, S., Sugawara, M., Watanabe, S. & Toyoshima, I. Identification of ER membrane targeting signal of kinectin. *Neurosci Lett* **413**, 238-240, doi:10.1016/j.neulet.2006.11.064 (2007).
72 Aurino, S. *et al.* Candidate-gene testing for orphan limb-girdle muscular dystrophies. *Acta Myol* **27**, 90-97 (2008).
73 Hibar, D. P. *et al.* Common genetic variants influence human subcortical brain structures. *Nature* **520**, 224-229, doi:10.1038/nature14101 (2015).
74 Mao, Q. *et al.* KTN1 Variants Underlying Putamen Gray Matter Volumes and Parkinson's Disease. *Front Neurosci* **14**, 651, doi:10.3389/fnins.2020.00651 (2020).
75 DeLong, M. R. *et al.* Role of basal ganglia in limb movements. *Hum Neurobiol* **2**, 235-244 (1984).
76 Alexander, G. E. & Crutcher, M. D. Preparation for movement: neural representations of intended direction in three motor areas of the monkey. *J Neurophysiol* **64**, 133-150, doi:10.1152/jn.1990.64.1.133 (1990).
77 Marchand, W. R. *et al.* Putamen coactivation during motor task execution. *Neuroreport* **19**, 957-960, doi:10.1097/WNR.0b013e328302c873 (2008).
78 Chen, S. *et al.* Neurotrimin expression during cerebellar development suggests roles in axon fasciculation and synaptogenesis. *J Neurocytol* **30**, 927-937, doi:10.1023/a:1020673318536 (2001).

79 Mazitov, T., Bregin, A., Philips, M. A., Innos, J. & Vasar, E. Deficit in emotional learning in neurotrimin knockout mice. *Behav Brain Res* **317**, 311-318, doi:10.1016/j.bbr.2016.09.064 (2017).
80 Li, C. *et al.* Genome-wide linkage and positional association analyses identify associations of novel AFF3 and NTM genes with triglycerides: the GenSalt study. *J Genet Genomics* **42**, 107-117, doi:10.1016/j.jgg.2015.02.003 (2015).
81 Brevik, E. J. *et al.* Genome-wide analyses of aggressiveness in attention-deficit hyperactivity disorder. *Am J Med Genet B Neuropsychiatr Genet* **171**, 733-747, doi:10.1002/ajmg.b.32434 (2016).
82 Cao, T. H. *et al.* Identification of novel biomarkers in plasma for prediction of treatment response in patients with heart failure. *Lancet* **385 Suppl 1**, S26, doi:10.1016/S0140-6736(15)60341-5 (2015).
83 Pan, Y., Wang, K. S. & Aragam, N. NTM and NR3C2 polymorphisms influencing intelligence: family-based association studies. *Prog Neuropsychopharmacol Biol Psychiatry* **35**, 154-160, doi:10.1016/j.pnpbp.2010.10.016 (2011).
84 Shende, P. & Desai, D. Physiological and Therapeutic Roles of Neuropeptide Y on Biological Functions. *Adv Exp Med Biol* **1237**, 37-47, doi:10.1007/5584_2019_427 (2020).
85 Diaz-delCastillo, M., Woldbye, D. P. D. & Heegaard, A. M. Neuropeptide Y and its Involvement in Chronic Pain. *Neuroscience* **387**, 162-169, doi:10.1016/j.neuroscience.2017.08.050 (2018).
86 Saraf, R., Mahmood, F., Amir, R. & Matyal, R. Neuropeptide Y is an angiogenic factor in cardiovascular regeneration. *Eur J Pharmacol* **776**, 64-70, doi:10.1016/j.ejphar.2016.02.033 (2016).
87 Southwick, S. M., Vythilingam, M. & Charney, D. S. The psychobiology of depression and resilience to stress: implications for prevention and treatment. *Annu Rev Clin Psychol* **1**, 255-291, doi:10.1146/annurev.clinpsy.1.102803.143948 (2005).
88 Nguyen, A. D., Herzog, H. & Sainsbury, A. Neuropeptide Y and peptide YY: important regulators of energy metabolism. *Curr Opin Endocrinol Diabetes Obes* **18**, 56-60, doi:10.1097/MED.0b013e3283422f0a (2011).
89 Autio, J., Stenback, V., Gagnon, D. D., Leppaluoto, J. & Herzig, K. H. (Neuro) Peptides, Physical Activity, and Cognition. *J Clin Med* **9**, doi:10.3390/jcm9082592 (2020).
90 Beck, B. & Pourie, G. Ghrelin, neuropeptide Y, and other feeding-regulatory peptides active in the hippocampus: role in learning and memory. *Nutr Rev* **71**, 541-561, doi:10.1111/nure.12045 (2013).
91 Kelly, S. A., Nehrenberg, D. L., Hua, K., Garland, T., Jr. & Pomp, D. Quantitative genomics of voluntary exercise in mice: transcriptional analysis and mapping of expression QTL in muscle. *Physiol Genomics* **46**, 593-601, doi:10.1152/physiolgenomics.00023.2014 (2014).
92 Kelly, S. A., Nehrenberg, D. L., Hua, K., Garland, T., Jr. & Pomp, D. Functional genomic architecture of predisposition to voluntary exercise in mice: expression QTL in the brain. *Genetics* **191**, 643-654, doi:10.1534/genetics.112.140509 (2012).
93 McGivney, B. A. *et al.* A genomic prediction model for racecourse starts in the Thoroughbred horse. *Anim Genet* **50**, 347-357, doi:10.1111/age.12798 (2019).
94 Hossain, M. I. *et al.* SULT4A1 Protects Against Oxidative-Stress Induced Mitochondrial Dysfunction in Neuronal Cells. *Drug Metab Dispos* **47**, 949-953, doi:10.1124/dmd.119.088047 (2019).
95 Garcia, P. L., Hossain, M. I., Andrabi, S. A. & Falany, C. N. Generation and Characterization of SULT4A1 Mutant Mouse Models. *Drug Metab Dispos* **46**, 41-45, doi:10.1124/dmd.117.077560 (2018).
96 Crittenden, F., Thomas, H. R., Parant, J. M. & Falany, C. N. Activity Suppression Behavior Phenotype in SULT4A1 Frameshift Mutant Zebrafish. *Drug Metab Dispos* **43**, 1037-1044, doi:10.1124/dmd.115.064485 (2015).

97 Chenaux, G. *et al.* Loss of SynDIG1 Reduces Excitatory Synapse Maturation But Not Formation In Vivo. *eNeuro* **3**, doi:10.1523/ENEURO.0130-16.2016 (2016).

98 Kalashnikova, E. *et al.* SynDIG1: an activity-regulated, AMPA- receptor-interacting transmembrane protein that regulates excitatory synapse development. *Neuron* **65**, 80-93, doi:10.1016/j.neuron.2009.12.021 (2010).

99 An, B. *et al.* Multiple association analysis of loci and candidate genes that regulate body size at three growth stages in Simmental beef cattle. *BMC Genet* **21**, 32, doi:10.1186/s12863-020-0837-6 (2020).

100 Erdenesan, E. *LIVESTOCK STATISTICS IN MONGOLIA*, <http://www.fao.org/fileadmin/templates/ess/documents/apcas26/presentations/APCAS-16-6.3.5_-_Mongolia_-_Livestock_Statistics_in_Mongolia.pdf> (2016).

101 Li, J. L., Shi, Y. F., Fan, C. Y. & Manglai, D. mtDNA Diversity and Origin of Chinese Mongolian Horses. *Asian Austral J Anim* **21**, 1696-1702, doi:DOI 10.5713/ajas.2008.80193 (2008).

102 Cosgrove, E. J. *et al.* Genome Diversity and the Origin of the Arabian Horse. *Sci Rep* **10**, 9702, doi:10.1038/s41598-020-66232-1 (2020).

103 Randhawa, I. A. S., Khatkar, M. S., Thomson, P. C. & Raadsma, H. W. Composite selection signals can localize the trait specific genomic regions in multi-breed populations of cattle and sheep. *Bmc Genetics* **15**, doi:Artn 34 10.1186/1471-2156-15-34 (2014).

104 QGIS.org. *QGIS Geographic Information System*. (QGIS Association, 2022).
