# Supplementary material for: Common protein-coding variants influence the racing phenotype in galloping racehorse breeds
Source: Commun Biol. 2022 Dec 13;5:1320. doi: 10.1038/s42003-022-04206-x (PMC9748125; doi:10.1038/s42003-022-04206-x)
Supplement: Supplementary file 3 — Description of Additional Supplementary Files [file 42003_2022_4206_MOESM3_ESM.pdf]

Description of Additional Supplementary Files

File name: Supplementary Data 1

Description: Ancestry contributions in each individual from four populations in an analysis of admixture using K=4.

File name: Supplementary Data 2

Description: Populations used for the Racing and non-Racing cohorts for the composite selection signals analysis.

File name: Supplementary Data 3

Description: Selection signals identified in all comparison cohorts (Racing versus non-racing, Arabian versus other breeds, Mongolian racing versus other breeds, Thoroughbred versus other breeds).

File name: Supplementary Data 4

Description: Exercise-relevant gene ontology terms enriched among genes in Racing selected regions.

File name: Supplementary Data 5

Description: 407 differentially expressed genes (DEGs) from exercise response (UE) transcriptome used as input for CSS SNP enrichment analysis.

File name: Supplementary Data 6

Description: 230 differentially expressed genes (DEGs) from training response (TR) transcriptome used as input for CSS SNP enrichment analysis.

File name: Supplementary Data 7

Description: Summary of the number of statistically significant SNPs pre- and post-data integration for Racing analysis.

File name: Supplementary Data 8

Description: Gene loci associated with enriched CSS SNPs in post-data integration for Racing analysis.

File name: Supplementary Data 9

Description: Summary of the number of statistically significant SNPs pre- and post-data integration for Mongolian Racing analysis.

File name: Supplementary Data 10

Description: Gene loci associated with enriched CSS SNPs in post-data integration for Mongolian Racing analysis.

File name: Supplementary Data 11

Description: Samples and population details for the horses used for whole genome resequencing.

File name: Supplementary Data 12

Description: Detailed information for the whole genome resequencing and alignment.

File name: Supplementary Data 13

Description: Summary of SNPs and INDELs in the 70 WGS.

File name: Supplementary Data 14

Description: Summary information for SNPs identified in this study and common site in MNEc2M SNP data.

File name: Supplementary Data 15

Description: Variants in candidate genes chosen for validation genotyping

File name: Supplementary Data 16

Description: Breeds used for tests of association between SNPs and racing

File name: Supplementary Data 17

Description: Allele frequencies for each SNP among the different breeds

File name: Supplementary Data 18

Description: Thoroughbred cohorts used for tests of association between SNPs and racing

File name: Supplementary Data 19

Description: Genetic association test results among the Thoroughbred cohorts
