# Supplementary material for: Common protein-coding variants influence the racing phenotype in galloping racehorse breeds
Source: Commun Biol. 2022 Dec 13;5:1320. doi: 10.1038/s42003-022-04206-x (PMC9748125; doi:10.1038/s42003-022-04206-x)
Supplement: Supplementary file 5 — Reporting Summary [file 42003_2022_4206_MOESM5_ESM.pdf]

Corresponding author(s): Emmeline Hill

Last updated by author(s): 09-Oct-2022

Reporting Summary

Nature Research wishes to improve the reproducibility of the work that we publish. This form provides structure for consistency and transparency in reporting. For further information on Nature Research policies, see our Editorial Policies and the Editorial Policy Checklist.

Statistics

For all statistical analyses, confirm that the following items are present in the figure legend, table legend, main text, or Methods section.

n/a	Confirmed	
☐	☒	The exact sample size (*n*) for each experimental group/condition, given as a discrete number and unit of measurement
☐	☒	A statement on whether measurements were taken from distinct samples or whether the same sample was measured repeatedly
☐	☒	The statistical test(s) used AND whether they are one- or two-sided<br>*Only common tests should be described solely by name; describe more complex techniques in the Methods section.*
☐	☒	A description of all covariates tested
☐	☒	A description of any assumptions or corrections, such as tests of normality and adjustment for multiple comparisons
☐	☒	A full description of the statistical parameters including central tendency (e.g. means) or other basic estimates (e.g. regression coefficient) AND variation (e.g. standard deviation) or associated estimates of uncertainty (e.g. confidence intervals)
☐	☒	For null hypothesis testing, the test statistic (e.g. *F*, *t*, *r*) with confidence intervals, effect sizes, degrees of freedom and *P* value noted<br>*Give P values as exact values whenever suitable.*
☒	☐	For Bayesian analysis, information on the choice of priors and Markov chain Monte Carlo settings
☐	☒	For hierarchical and complex designs, identification of the appropriate level for tests and full reporting of outcomes
☒	☐	Estimates of effect sizes (e.g. Cohen's *d*, Pearson's *r*), indicating how they were calculated

Our web collection on statistics for biologists contains articles on many of the points above.

Software and code

Policy information about availability of computer code

Data collection	No software was used for data collection.
Data analysis	To visualise genetic relatedness among the populations, principal component analysis (PCA) was performed using smartPCA from the EIGENSOFT package (version 4.2). For the analysis of population substructure, model-based clustering was performed using the software package ADMIXTURE. Composite selection signals (CSS) analyses were performed to investigate genomic signatures of selection. gwinteR was used to integrate the SNP data arising from the CSS analyses with gene sets generated from functional genomics data analyses. Paired-end reads were mapped to the reference genome using BWA (Burrows-Wheeler Aligner) (Version: 0.7.8). Duplicated reads were removed using SAMtools. SNP calling was performed using both SAMtools and GATK v4.1.2.0. GATK SelectVariants was used to select a common subset of variants between unfiltered GATK and SAMtools calling result. Tests of genetic association were performed in PLINK v1.9 by comparing allele frequencies in a chi-square (1df) test for the binary traits, and a Wald test for the quantitative trait.

For manuscripts utilizing custom algorithms or software that are central to the research but not yet described in published literature, software must be made available to editors and reviewers. We strongly encourage code deposition in a community repository (e.g. GitHub). See the Nature Research guidelines for submitting code & software for further information.

Data

Policy information about availability of data

All manuscripts must include a data availability statement. This statement should provide the following information, where applicable:

- Accession codes, unique identifiers, or web links for publicly available datasets
- A list of figures that have associated raw data
- A description of any restrictions on data availability

SNP array derived genotypes generated in this study have been deposited in the European Variation Archive with the accession IDs PRJEB55561 (Project),

ERZ12817059 (Mongolian horse analysis), and ERZ12817060 (Arabian horse analysis). The whole genome sequence data have been deposited in the Sequence Read Archive with the BioProject ID: PRJNA867509. The source data for Figure 2 is available at Source data is available at doi:10.5061/dryad.g79cnp5sm. The SNP genotype data generated for the validation study are subject to the following licenses/restrictions: The phenotype and genotype data are the property of Plusvital Ltd. and subject to a confidentiality agreement with the animal owners.

Field-specific reporting

Please select the one below that is the best fit for your research. If you are not sure, read the appropriate sections before making your selection.

☐ Life sciences ☐ Behavioural & social sciences ☒ Ecological, evolutionary & environmental sciences

For a reference copy of the document with all sections, see nature.com/documents/nr-reporting-summary-flat.pdf

Ecological, evolutionary & environmental sciences study design

All studies must disclose on these points even when the disclosure is negative.

Study description	This is a population genomics analysis identifying genetic variants contributing to the racing phenotype in horse breeds. A cohort based approach was used. We identified genomic regions of interest by scanning for selection signals for racing. We integrated the SNPs from the population genomics analyses with differentially expressed gene sets from equine skeletal muscle to prioritise genes. We generated whole genome sequence data for Asian horses to catalogue genetic variation. We screened for putative protein-coding variants in candidate genes. To validate the variants for racing, we performed genetic association analyses in subsets of Thoroughbred and Mongolian horses and in a range of other racing and non-racing breeds.
Research sample	Our analyses are based on genotype data from more than 1600 domestic horses from a range of different global breeds.
Sampling strategy	Samples were collected for 1) commercial genetic testing over a period of 15 years and 2) whole genome sequencing from Asian samples over a period of 5 years.
Data collection	Commercial samples were submitted for genetic testing. Where horse IDs were provided, racing phenotype data was accessed and recorded in a database. The Asian samples were collected in the field and breed assignment and country of origin were recorded.
Timing and spatial scale	Samples were collected over a period of 15 years from Europe, North America, Australasia and Asia.
Data exclusions	One outlier (Shire, ID:SH144) was identified in the PCA and excluded from further analyses. The outlier was separated from the breed cluster and had also been identified as an outlier in the study from which the data were obtained
Reproducibility	Tests of association in independent validation samples demonstrate reproducibility
Randomization	Samples were allocated according to 1) breed, 2) racing phenotype or non-racing phenotype or 3) elite racing Thoroughbred or non-elite racing Thoroughbred
Blinding	No blinding

Did the study involve field work? ☒ Yes ☐ No

Field work, collection and transport

Field conditions	Dry, moderate temperature
Location	Khentii Provence, Mongolia.
Access & import/export	Horse tail hair samples were obtained with owner's consent, and with the cooperation and assistance of the owner's horse handlers (herdsmen). Samples were shipped directly to Ireland for commercial genetic testing with associated export/import licences obtained. Horse hairs that were not used for DNA extraction have been preserved in an urn and maintained at Plusvital's offices in Ireland.
Disturbance	Horses were herded and corralled in their own habitat, with minimal alteration to routine handling.

Reporting for specific materials, systems and methods

We require information from authors about some types of materials, experimental systems and methods used in many studies. Here, indicate whether each material, system or method listed is relevant to your study. If you are not sure if a list item applies to your research, read the appropriate section before selecting a response.

Materials & experimental systems

n/a	Involved in the study
☒	☐ Antibodies
☒	☐ Eukaryotic cell lines
☒	☐ Palaeontology and archaeology
☐	☒ Animals and other organisms
☒	☐ Human research participants
☒	☐ Clinical data
☒	☐ Dual use research of concern

Methods

n/a	Involved in the study
☒	☐ ChIP-seq
☒	☐ Flow cytometry
☒	☐ MRI-based neuroimaging

Animals and other organisms

Policy information about studies involving animals; ARRIVE guidelines recommended for reporting animal research

Laboratory animals	This study did not involve laboratory animals.
Wild animals	This study did not involve wild animals.
Field-collected samples	This study involved samples collected from the field (Mongolian Racing)
Ethics oversight	Samples genotyped in this study were collected with informed owner's consent for commercial genetic testing and approved for use in research. As such, institutional animal research ethics was not required. Approval for collection and the movement of genetic material for the research was granted by Inner Mongolia Agricultural University Animal Research Ethics Committee and Mongolian University of Science and Technology.

Note that full information on the approval of the study protocol must also be provided in the manuscript.
